# Health-related quality of life and physical activity in children with inherited cardiac arrhythmia or inherited cardiomyopathy: the prospective multicentre controlled QUALIMYORYTHM study rationale, design and methods

**DOI:** 10.1186/s12955-021-01825-6

**Published:** 2021-07-28

**Authors:** Pascal Amedro, Oscar Werner, Hamouda Abassi, Aymeric Boisson, Luc Souilla, Sophie Guillaumont, Johanna Calderon, Anne Requirand, Marie Vincenti, Victor Pommier, Stefan Matecki, Gregoire De La Villeon, Kathleen Lavastre, Alain Lacampagne, Marie-Christine Picot, Constance Beyler, Christophe Delclaux, Yves Dulac, Aitor Guitarte, Philippe Charron, Isabelle Denjoy-Urbain, Vincent Probst, Alban-Elouen Baruteau, Philippe Chevalier, Sylvie Di Filippo, Jean-Benoit Thambo, Damien Bonnet, Jean-Luc Pasquie

**Affiliations:** 1 Department of Paediatric and Adult Congenital Cardiology, M3C National Reference Centre, Haut-Lévêque Cardiology Hospital, Bordeaux University Hospital, Avenue de Magellan, 33604 Pessac Cedex, France; 2 INSERM, Bordeaux Cardio-Thoracic Research Centre, U1045, University of Bordeaux, Pessac, France; 3 IHU Liryc, Electrophysiology and Heart Modelling Institute, Fondation Bordeaux Université, Pessac, France; 4 Paediatric and Congenital Cardiology Department, M3C Regional Reference CHD Centre, Montpellier University Hospital, Montpellier, France; 5 Paediatric Cardiology and Rehabilitation Unit, Institut-Saint-Pierre, Palavas-Les-Flots, France; 6 Epidemiology and Clinical Research Department, Montpellier University Hospital, Montpellier, France; 7 Paediatric Cardiology and Physiology Department, Robert Debré University Hospital, University of Paris, AP-HP, Paris, France; 8 Paediatric Cardiology Department, M3C Regional Reference Centre, Toulouse University Hospital, Toulouse, France; 9 Department of Cardiology, National Reference Centre for Inherited Cardiomyopathy, University of Paris, AP-HP, Paris, France; 10 Department of Cardiology, National Reference Centre for Inherited Cardiac Arrhythmia, L’institut du thorax, INSERM, CNRS, University of Nantes, Nantes University Hospital, Nantes, France; 11 Department of Pediatric Cardiology and Pediatric Cardiac Surgery, L’Institut du Thorax, INSERM, CNRS, University of Nantes, Nantes University Hospital, Nantes, France; 12 Department of Congenital Cardiology, National Reference Centre for Inherited Cardiac Arrhythmia, University of Lyon, Lyon University Hospital, Lyon, France; 13 Paediatric Cardiology Department, Necker-Enfants malades, M3C National Reference Centre, University of Paris, AP-HP, Paris, France; 14 Cardiology Department of Cardiology, Regional Reference Centre for Inherited Cardiac Arrhythmia, Montpellier University Hospital, Montpellier, France; 15 PhyMedExp, INSERM, CNRS, University of Montpellier, Montpellier, France

**Keywords:** Quality of life, Physical activity, Paediatrics, Inherited cardiac arrhythmia, Genetic cardiomyopathy

## Abstract

**Background:**

Advances in paediatric cardiology have improved the prognosis of children with inherited cardiac disorders. However, health-related quality of life (QoL) and physical activity have been scarcely analysed in children with inherited cardiac arrhythmia or inherited cardiomyopathy. Moreover, current guidelines on the eligibility of young athletes with inherited cardiac disorders for sports participation mainly rely on expert opinions and remain controversial.

**Methods:**

The QUALIMYORYTHM trial is a multicentre observational controlled study. The main objective is to compare the QoL of children aged 6 to 17 years old with inherited cardiac arrhythmia (long QT syndrome, Brugada syndrome, catecholaminergic polymorphic ventricular tachycardia, or arrhythmogenic right ventricular dysplasia), or inherited cardiomyopathy (hypertrophic, dilated, or restrictive cardiomyopathy), to that of age and gender-matched healthy subjects. The secondary objective is to assess their QoL according to the disease’s clinical and genetic characteristics, the level of physical activity and motivation for sports, the exercise capacity, and the socio-demographic data. Participants will wear a fitness tracker (ActiGraph GT3X accelerometer) for 2 weeks. A total of 214 children are required to observe a significant difference of 7 ± 15 points in the PedsQL, with a power of 90% and an alpha risk of 5%.

**Discussion:**

After focusing on the survival in children with inherited cardiac disorders, current research is expanding to patient-reported outcomes and secondary prevention. The QUALIMYORYTHM trial intends to improve the level of evidence for future guidelines on sports eligibility in this population.

*Trial registration* ClinicalTrials.gov Identifier: NCT04712136, registered on January 15th, 2021 (https://clinicaltrials.gov/ct2/show/NCT04712136).

## Background

Prognosis of children with congenital cardiac disorders has significantly improved in the past two decades, due to great advances in prenatal screening, intensive neonatal care and modern invasive therapies [1]. As a result, more attention has been recently given to health-related quality of life (QoL) and secondary prevention in paediatric cardiology [2, 3].

Traditionally, congenital cardiac disorders are divided into 3 groups: congenital heart disease (CHD) for structural cardiac anomalies, inherited cardiac arrhythmia and inherited cardiomyopathy. So far, QoL studies in paediatric cardiology have mainly focused on the group of children with CHD and, although the overall level of QoL is good in this population, our group highlighted the existing correlation between exercise capacity and QoL, and the risk for physical deconditioning, even for non-serious CHD [4, 5]. Indeed, despite the promotion of physical activity in the current guidelines [6–8], many children with CHD experience social barriers to physical activity and “remain on the side-line” at school or in their social life [9]. Ultimately, these patients may be exposed to the consequences of a sedentary lifestyle: overweight, high blood pressure, social exclusion, and impaired QoL [10–12]. Conversely, patients with CHD who have been physically active since childhood are less likely to become sedentary adults [13].

Such consistent data are not available in the literature for children from the two other groups, e.g. inherited cardiac arrhythmia or inherited cardiomyopathy, for which QoL and physical activity have been rarely studied. Some heterogeneous data were provided from small paediatric [14–16] or adult [17, 18] cohorts, however no prospective controlled study has analysed the determinants of QoL and the level of physical activity in children with inherited cardiac disorders [19]. We hypothesized that QoL scores and physical activity levels in children with inherited cardiac disorders are reduced compared to the healthy subjects.

Therefore, the multicentre observational controlled QUALIMYORYTHM trial aims to evaluate the QoL, exercise capacity, and the level of physical activity of children with inherited cardiac arrhythmia or inherited cardiomyopathy, in comparison with a matched control population. This study will also evaluate the main factors associated with QoL in this population.

## Methods

### Objectives

The main objective of the QUALIMYORYTHM trial is to compare the QoL of children aged 6 to 17 years old with inherited cardiac arrhythmia or inherited cardiomyopathy to that of matched healthy children.

The secondary objective is to assess, in this population, the QoL according to the disease clinical and genetic characteristics, the level of physical activity, the exercise capacity, and the socio-demographic data.

### Study design

**Fig. 1:**
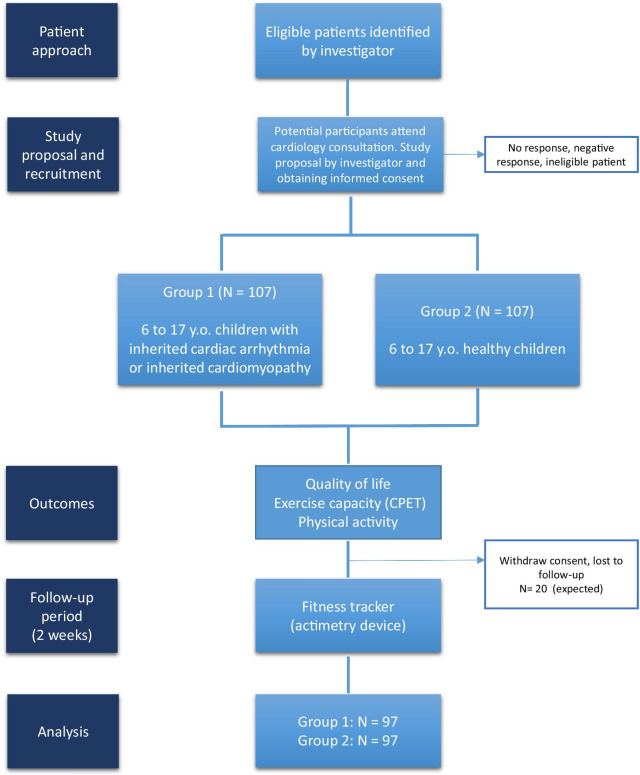
Flow chart

The QUALIMYORYTHM trial is a prospective, multicentre controlled observational study with a follow-up of 2 weeks and a recruitment duration of 18 months. All screened subjects will be identifiable throughout the study by a unique subject number. The eligible patients will be divided into 2 groups (Fig. 1):

Group 1: children aged 6 to 17 years old with an inherited cardiac arrhythmia or an inherited cardiomyopathy.

Group 2: age and gender-matched control group, with healthy children aged 6 to 17 years old.

### Setting

Patients will be recruited during a paediatric cardiology consultation among the French expert centres for inherited cardiac arrhythmia or an inherited cardiomyopathy (CARDIOGEN national network, http://www.filiere-cardiogen.fr). Overall, 7 university hospitals, labelled as expert centres in paediatric inherited cardiac disorders by health authorities, will participate in patient recruitment.

Conduct of the study will be led by the principal investigator (supported, when necessary, by the co-investigators), a research fellow, and a clinical research coordinator, all of whom are trained in Good Clinical Practice and in the requirements of the study protocol.

### Study population

Children aged of 6 to 17 years old with an inherited cardiac arrhythmia (long QT syndrome, Brugada syndrome, catecholaminergic polymorphic ventricular tachycardia, or arrhythmogenic right ventricular dysplasia), or those with an inherited cardiomyopathy (hypertrophic, dilated, or restrictive cardiomyopathy), will be prospectively recruited during their annual follow-up.

The age- and gender-matched control group will be defined using the same method as in our previous paediatric exercise test studies [4, 5, 20–22]. Children aged 6 to 17 years old referred to the paediatric cardiology consultation for a non-severe functional symptom linked to exercise (murmur, palpitation, or dyspnoea) or for a medical sports certificate will be assessed for eligibility. These children will be classified in the control group only after a completely normal check-up, including physical examination, electrocardiogram, and echocardiography. Children with any chronic disease, medical condition (cardiac, neurologic, respiratory, muscular, or renal), or medical treatment and those requiring any further specialized medical consultation will not be eligible.

Patients who are not able to answer the quality of life questionnaire (e.g. non-French speakers, severe mental illness) will not be eligible for the study. Detailed inclusion and exclusion criteria are reported in Table 1.

**Table 1:** Trial entry

Inclusion criteria
Male or female aged 6 to 17 years old
Group 1: Patients with an inherited cardiac arrhythmia (long QT syndrome, Brugada syndrome, catecholaminergic polymorphic ventricular tachycardia, or arrhythmogenic right ventricular dysplasia), or those with an inherited cardiomyopathy (hypertrophic, dilated, or restrictive cardiomyopathy) Group 2: Children with a completely normal check-up, referred to the paediatric cardiology consultation for a non-severe functional symptom linked to exercise (murmur, palpitation, or dyspnoea) or for a medical sports certificate
Informed consent of parents or legal guardians, and oral assent of children
Exclusion criteria
Patients who are not able to understand or fill out the questionnaires (QoL, physical activity and motivation questionnaires) Absolute contraindications for CPET: fever, uncontrolled asthma, respiratory failure, acute myocarditis or pericarditis, uncontrolled arrhythmias causing symptoms or haemodynamic compromise, uncontrolled heart failure, acute pulmonary embolus or pulmonary infarction, and children with mental impairment leading to inability to cooperate Group 2: Children with any chronic disease, medical condition (cardiac, neurologic, respiratory, muscular, or renal), or medical treatment and those requiring any further specialized medical consultation

### Primary outcome

The primary outcome is the total QoL score with the self-reported PedsQL questionnaire. The PedsQL questionnaire has four multidimensional scales: physical functioning (8 items), emotional functioning (5 items), social functioning (5 items), and school functioning (5 items). The three summary scores are: total scale score (23 items), physical health summary score (8 items), psychosocial health summary score (15 items). Each item uses a 5-point Likert scale from 0 (never) to 4 (almost always). Items are reversed scored and linearly transformed to a 0–100 scale, higher scores indicating a better QoL. This instrument was validated by Varni et al. in healthy and patient populations and its psychometric properties showed reliability, validity and responsiveness to clinical change over time [23]. The translation and cultural adaptation into French was performed by MAPI Research Institute (www.mapi-trust.org), following the international guidelines [23] and this version showed good psychometric properties [24]. We recently performed the complete psychometric validation of the French self and proxy versions of the PedsQL generic questionnaire for 8 to 12 year-old children [25]. We also found a good sensitivity of the PedsQL in several controlled prospective HRQoL studies among healthy controls and children with various chronic diseases [26–29].

In this study, three age versions of the PedsQL questionnaire will be used (e.g. 5–7, 8–12 and 13–17 years old). Parents and children will complete the QoL questionnaires during the routine paediatric cardiology consultation (inclusion visit). Children aged 8 years old and above will complete the PedsQL self-questionnaire under trained nurse supervision, as in our similar QoL studies [26–29].

### Secondary outcomes

- The level of physical activity will be assessed by:
  - The metabolic equivalent of task (MET), measured by a fitness tracker (ActiGraph GT3X accelerometer, Pensacola, FL, USA) [30]. The MET is defined as the ratio of physical activity to basal metabolic demand. The metabolic equivalence scale ranges from 0.9 MET (at rest) to 18 MET (run at 17.5 km/h). The fitness tracker will be assigned during the inclusion visit and the participants will be instructed to wear it at the waist at all times for 14 days, except during sleep and water-based activities such as swimming or bathing.
  - The Ricci and Gagnon physical activity questionnaire, composed by 8 items (total score < 16 points: no activity; 17 to 32 points: moderate activity; 33 to 40 points: intensive activity) will be completed during the inclusion visit by the participant. Children aged 8 years old and above will complete this questionnaire under trained nurse supervision [31].
  - The EMAPS questionnaire (motivation scale for physical activity in a health context), a 18-item motivation scale towards health-oriented physical activity recently validated in French [32]. This questionnaire will be completed during the inclusion visit by the participant. Children aged 8 years old and above will complete this questionnaire under trained nurse supervision.
- The exercise capacity will be assessed by a cardio-pulmonary exercise test (CPET), using the same methodology as in our previous paediatric CPET studies [5, 20, 21, 33]. The following variables will be measured: maximum oxygen uptake (VO2_max_); ventilatory anaerobic threshold (VAT); ventilatory efficiency (VE/VCO2 slope), oxygen uptake efficiency slope (OUES), and maximum oxygen pulse (VO2_max_/maximum heart rate). Exercise test procedures in all participating laboratories will be harmonized before the start of the study. All centres will use the same CPET cycle ergometer protocol, to obtain a homogeneous incremental overall duration between 8 to 12 min: a 1-min rest; a 3-min warm-up (10 to 20 watts) in increments of 10, 15, or 20 watts each minute; a pedalling rate of 60 to 80 revolutions per minute; a 3-min active recovery (20 watts); and a 2-min rest. The CPET will be considered as maximal when 3 out of the 4 following criteria will be reached: respiratory exchange ratio (RER = VCO2/VO2) ≥ 1, maximum heart rate > 85% of maximal age-predicted heart rate, limit of the patient’s tolerance despite verbal encouragement, plateau of VO2 (VO2_max_) despite the increasing exercise intensity, and patient's inability to provide a minimum pedalling frequency of 60 per minute despite verbal encouragement. The VAT will be estimated using Beaver’s method [34]. VO2_max_ and VAT values will be normalized in a percentage of the predicted VO2_max_ using reference values for cycle ergometer test in the general paediatric population [35, 36]. When the VO2_max_ does not reach criteria for maximal effort, the peak VO2 will be informed, as usual in paediatrics [37, 38].
- The parents-reported QoL scores will be assessed by the proxy questionnaires of the PedsQL (e.g. 5–7, 8–12 and 13–17 y.o. proxy versions) [23]. Both parents will be able to participate and will therefore separately fill out a PedsQL proxy questionnaire.

All outcomes are reported in Table 2.

**Table 2:** Outcome measures

Primary outcome
Health-related quality of life score: PedsQL self-questionnaire (versions 5–7 years and 8–12 years for children and version 13–17 years for adolescents)
Secondary outcomes
Level of physical activity assessed by: The metabolic equivalent of task (MET) assessed by the ActiGraph GT3X accelerometer The Ricci and Gagnon physical activity questionnaire The EMAPS motivation questionnaire
CPET variables: VO2_max_ (maximum oxygen uptake) VAT (ventilatory anaerobic threshold) VE/VCO2 slope (ventilarory efficiency) OUES (oxygen uptake efficiency slope) Maximum oxygen pulse (VO2_max_/maximum heart rate)
Health-related quality of life score: PedsQL proxy questionnaire (versions 5–7 years and 8–12 years for children and version 13–17 years for adolescents)

### Other data collection

- Clinical outcomes: age at diagnosis (prenatal, postnatal), genetic anomaly, NYHA functional class, blood pressure, body mass index (BMI), healthcare usage (primary and secondary care contacts, hospitalisation), pacemaker, implantable cardioverter defibrillator (ICD), cardiac surgery, and medication.
- Socio-economic status of the patient’s family.
- Safety outcomes.

### Sample size

We aim to recruit a total of 214 children in both groups. The primary outcome is the difference in the self-reported QoL score with the PedsQL instrument between the children with inherited cardiac disorders and the control group. We used the results from our previous QoL cross-sectional studies in patients with CHD to calculate the sample size [4, 21, 26–29, 39]. In our experience, as well as in similar studies using patient related outcomes, a difference of less than 5 points is not clinically relevant, and a difference of more than 10 points is ideal, but rarely achieved [2, 27, 29]. Therefore, we hypothesized to observe a difference in the total QoL score of 7 ± 15 points (over 100). With a 90% power, a bilateral alpha risk of 5%, and potentially 10% of loss to follow-up or missing data on the primary outcome, we need to include 107 patients in the group 1 and 107 patients in the group 2.

### Statistical analysis

Patients’ characteristics will be presented using mean and standard deviation for continuous variables, and frequencies and proportions for categorical variables. QoL scores will be calculated with the parametric Student’s t test when the distribution was Gaussian and with the Mann–Whitney test otherwise. The effect size will be estimated using Cohen’s d measure.

Association between QoL scores and physical activity, clinical and socio-demographic data will be studied using Pearson’s or Spearman’s coefficient. According to the type of relation between QoL scores and these factors, a multiple linear regression or a generalized additive model (GAM) will be implemented for the different scores. In the GAM, a parametric portion of the model could be identified (with the vector of parameters associated with the set of explanatory variables). The non-parametric part of the model could be formed by smooth functions for clinical characteristics or age effects for example. One of the main advantages of the GAM implemented is the possibility to address the issue of linear dependency (i.e. collinearity) between these characteristics or age effects which often impedes their simultaneous estimation.

When considering multiple comparisons, the type I error will be adjusted.

The statistical significance will be set at 0.05 and analyses will be performed using Statistical Analysis Systems version 9 (SAS Institute, Cary, NC, USA). Data will be managed by the clinical research unit of Montpellier University Hospital, France, in collaboration with the paediatric and congenital cardiology department.

### Ethics

The study will be conducted in compliance with the Good Clinical Practices protocol and Declaration of Helsinki principles. It was approved by a drawn national Ethics Committee (CPP Sud-Est VI, 2020-A00411-38). Informed consent will be obtained from all parents or legal guardians, and oral assent will be obtained from all children. The study was registered on Clinicaltrials.gov (NCT04712136).

## Expected results and perspectives

In the continuity of our research program on QoL and physical activity in paediatric and congenital cardiology [4, 21, 22, 26, 26–29, 39], the QUALIMYOTYRHM trial should provide, for the first time, reliable data on health-related quality of life and physical activity in children with inherited cardiac arrhythmia or inherited cardiomyopathy. From our perspective, considering the QoL as the main outcome of this trial is both original and primordial in this young population with cardiac inherited disorders. Indeed, measuring patient-reported outcomes (PRO) in paediatric cardiology should become a priority, considering that many young cardiac patients experience a low level of physical impairment during childhood, but an increased cardio-vascular risk during adulthood [2, 3, 40]. This is also in line with the European Medicines Agency (EMA) promotion of PRO measures in clinical research, and more and more drug trials, even in paediatrics, currently evaluate the quality of life as a secondary outcome [41]. Moreover, our results will contribute to provide consistent baseline clinical and QoL data for future interventional clinical trials in children with inherited cardiac arrhythmia or inherited cardiomyopathy.

The QUALIMYORYTHM trial also seeks to determine the level of physical activity in the contemporary generation of children with inherited cardiac disorders, using modern connected technology adapted to this population, such as fitness trackers. In the absence of any reliable risk stratification in those children, the current guidelines on eligibility of young athletes with inherited cardiac disorders for sports participation mainly rely on expert opinions [42–45], and conclusions by the experts have classically not been the same on both sides of the Atlantic [46]. The difference commonly made between recreational and competitive sport is probably more about medico-legal issues for the practitioner, than for the patient's own interest. For example, more than a decade ago, the European guidelines for competitive sport participation in the long QT syndrome were very restrictive [43]. Yet, in a recent study from a cohort of 172 appropriately managed children with long QT syndrome, cardiac event rates were low and occurred during recreational, but not competitive activities [47]. Similarly, in a cohort of 129 young athletes with ICD, most of them having a long QT syndrome or a hypertrophic cardiomyopathy, and participating in competitive or dangerous sports, there were no occurrences of death, arrest, or injury related to arrhythmia, during sports; interestingly, this study found that 27% patients received at least one shock, only 4 during sports, which is similar to unselected non-athletic paediatric ICD cohorts [48]. Based on convergent studies reporting a low risk of cardiac event in athletes with optimal treatment for long QT syndrome [47–50], a progressive trend towards a different medical approach has been observed, involving the patient-athlete in shared decision-making. Therefore, the 2015 US guidelines [51] have been less restrictive regarding high intensity recreational exercise and competitive sports, especially for asymptomatic genotype-positive/phenotype-negative athletes. More recently, the 2020 European guidelines have moved one step further in promoting supervised and regular physical activity in patients with inherited cardiac disorders [45].

Nevertheless, most published studies are retrospective and have not evaluated sports with the highest cardiovascular demand (only a few class IIIB and IIIC sports), or recreational sports in children with the rarest or most serious inherited cardiac disorders. Indeed, in their recent review, Masrur et al. concluded that there are insufficient data on the risks of exercise in Brugada syndrome to make recommendations for exercise [52]. Therefore, the QUALIMYORYTHM trial should contribute to precise how the recommendations are currently applied “in the real life”, and measure their potential impact on QoL and physical activity in children with inherited cardiac arrhythmia or inherited cardiomyopathy.

Furthermore, the QUALIMYORYTHM trial will provide, for the first time in a population of children with inherited cardiac disorders, consistent cardiopulmonary fitness data, such as the maximum oxygen uptake (VO2_max_), the anaerobic threshold, the ventilatory efficiency, and the exercise stroke volume. Moreover, this study will also bring original information about the level of motivation of children with inherited cardiac disorders for physical activity, and its degree of correlation with the actual levels of physical activity and exercise capacity. In paediatric cardiology, cardiopulmonary fitness has mainly been determined in children with CHD [5, 53], but these results cannot be easily transposed to inherited cardiac disorders, as the underlying pathophysiology and haemodynamic consequences are different. For instance, animal models have demonstrated that the beneficial effects of training on the heart effects of training are related to the signalling pathways of myocardial hypertrophy and fibrosis, but such findings need to be further analysed in patients with inherited cardiomyopathy [54].

If the QUALIMYORYTHM trial finds that physical activity and exercise capacity in children with inherited cardiac arrhythmia or inherited cardiomyopathy are similar to those of healthy children, without any additional risk, the level of evidence for future guidelines on sports eligibility in this population will be significantly improved. Conversely, if physical activity and exercise capacity in children with inherited cardiac disorders are significantly impaired, it will open a new research field on rehabilitation programs in this population. Indeed, if physical deconditioning is diagnosed and managed at an early stage in patients with inherited cardiac disorders, participation in rehabilitation programs could reverse the vicious circle of deconditioning, and, ultimately participate in reducing the cardio-vascular risks related to inactivity, as it has been suggested in CHD [22, 55–57], as well as in many chronic diseases [58].

### Study limitations

The subjects in the control group will be recruited at the hospital and may not be considered as healthy as if they were recruited from the general population. However, this bias was limited in our previous similar studies, which showed very good correlations between QoL scores and VO2_max_ values with the respective normal values reported in the literature among the general population [5, 39].

## Data Availability

Not applicable.

## Abbreviations

CHD: Congenital heart disease
CPET: Cardiopulmonary exercise test
QoL: Health-related quality of life
